# Representing the Public in Public Engagement: The Case of the 2008 UK Stem Cell Dialogue

**DOI:** 10.1371/journal.pbio.1001418

**Published:** 2012-11-13

**Authors:** Alison Mohr, Sujatha Raman

**Affiliations:** Institute for Science and Society, School of Sociology and Social Policy, University of Nottingham, Nottingham, United Kingdom; Kings College London, United Kingdom

## Abstract

Engaging the public as architects, rather than simply as subjects or objects, of the substantive content of interactive dialogue may help fulfill the democratic potential of public engagement.

Efforts to engage the public in science take many forms, yet in many cases, “engagement” is a means toward acceptance rather than true participation. In 2008, the largest ever public engagement (PE) exercise sponsored by UK Research Councils was held. The Stem Cell Dialogue (SCD), designed to identify the range of views and concerns amongst the wider public about stem cell research, was jointly supported by the Biotechnology and Biological Sciences Research Council (BBSRC), the Medical Research Council (MRC), and Sciencewise. The SCD revealed high levels of public support for stem cell science and technology, according to the official press release [1], and thus seems to validate the traditional reasons offered for conducting PE around cutting-edge science: that engaging the wider public in dialogue at an early stage can help scientists communicate the motivations for their research, including its expected societal benefits, assuage potential ethical concerns, avert damaging controversies, and secure public acceptance. But, is this *instrumental* rationale—engagement toward a predetermined goal—sufficient? Can it offer the democratic legitimacy that underlies the recent turn to this type of “upstream” engagement? And does the SCD as it actually unfolded merit the summary finding of public support reported in the press release? In this paper, we draw from our work as official evaluators of the SCD (see Box 1), and recent debates on the purpose of engagement, to ask: how should we understand the “public” in PE; why is PE important for both society and science; and what lessons should we take from actual PE exercises?

**Box 1. Evaluation Methodology**

Our evaluation utilised a multi-method approach combining documentary analysis of project materials and scoping documents commissioned by BBSRC/MRC, participant questionnaires, structured observation of the workshops, and semi-structured interviews with seven Oversight Group members (chosen to represent a cross-section of the group and for their continuous involvement) and the three lead dialogue facilitators to explore their assessments of the dialogue in the context of its objectives and of the evaluation criteria. Of 569 questionnaires distributed at the workshops, 208 were returned, giving a response rate of over one-third (36.6%). Questionnaires were coded to enable the matching of responses across the sequence and location of workshops.

Two evaluators observed 11 workshops to cover the range of locations and sequence of workshops. Detailed observation notes were taken using an observation protocol adapted from Horlick-Jones et al. [10] that drew attention to the various activities and outputs and whether these could be considered successful against the normative evaluation criteria. Observers also recorded their broader impressions of different aspects of the events. Observer bias was limited by adherence to the protocol and the comparison of data from workshops attended by both evaluators. Quantitative data were analysed using SPSS and qualitative data were analysed in accordance with the evaluation criteria.

The evaluation was constrained in a number of ways. First, the scale of the budget and the number of person days stipulated by the sponsors precluded a highly detailed evaluation exercise of the extent that would seem appropriate to the sponsors' objectives, especially with regard to longer-term impact. Second, the evaluators were not consulted on their availability to attend the various workshops and were thus reliant on the facilitators, in some instances, to distribute the questionnaires. This meant that we could not be sure that the purpose, importance, and independence of the evaluation were effectively communicated. Third, while the evaluation brief specified access to the public participants for interviews, access was restricted due to the confidentiality/privacy agreements BMRB had with the participants.

## Which “Public” and What “Engagement”?

How the “public” is defined in various initiatives depends on the rationale for asking for public input. This in turn affects how members of the public are brought together and represented.

Previous articles in this series [2],[3] have identified three rationales for PE [4] and have critiqued the most common *instrumental* rationale to enhance public trust in novel areas of science and acceptance of the future technologies or to legitimise research policy decisions. The value of public dialogue in a democratic society is twofold. From a *normative* perspective, the process of PE is in itself a good thing in that the public should be consulted on decisions in which they have a stake. From a *substantive* standpoint, PE generates manifold perspectives, visions, and values that are relevant to the science and technologies in question, and could potentially lead to more socially robust outcomes (which may differ from the outcomes envisaged by sponsors or scientists).

Yet in any PE exercise, challenges arise not just in bringing people together, but also in facilitating interactions to ensure that different perspectives are elicited and considered and that the outcome provides a legitimate picture of public dialogue. For PE around bioscience developments to conform to democratic ideals, it has been asserted, participants must be broadly representative of the “affected public” [5]. This concept of *representativeness* forms a key normative criterion of a widely used framework for evaluating PE effectiveness [5]. Representativeness refers to the degree to which participants embody the socio-demographic characteristics of the affected population, including the relative distribution of views. However, representativeness of participation—that is, the diversity of opinions expressed—may not necessarily translate to representativeness in the end. In practice, a diversity of outcomes is often inhibited by the particular method, process, and reporting of engagement, which thus may lead to failure in democratic terms.

In the translation from theory to practice, rationales for PE (explicit or implicit) may change or become blurred, undercutting transparency or legitimacy [6]. Such changes can lead to tensions in the practice of PE that may affect the ways in which the “representativeness” of the public is construed. These rationales provide a useful framework for thinking about just how representative a given public may be under different scenarios. The SCD illustrates how competing rationales for representing the public can lead to particular outcomes that conflict with the democratic ideals of PE.

## Stem Cell Dialogue

An oversight sroup comprised of 19 members representing a broad range of interests relating to stem cell research—including from universities, charities, and public interest groups—commissioned the British Market Research Bureau (BMRB) to deliver the UK-wide public dialogue. BMRB developed a deliberative process that brought together members of the public with scientists, clinicians, social scientists, and ethicists. A total of 15 workshops—three each in London, Bristol, Cardiff, Edinburgh, and Newcastle—were held, beginning with an introduction to stem cells and moving on to discussions of social and ethical issues around sourcing of stem cells, potential future applications, clinical trials, and stem cell banks.

The sponsors' official aims and objectives embodied both a normative commitment to “engage the *diverse public* about developments in stem cell research, to account for their views in policy development” and a substantive aspiration to “identify the *range of views and concerns* about the science and ethics of stem cell research amongst the wider public and their societal context” (authors' emphasis). Yet the twin goals of diversity of participation and perspectives failed to translate, in practice, into diversity of outcomes—the officially reported outcome being high levels of public support for stem cell science and technology. Even BMRB's own report had noted this result, though the document downplayed it by outlining various ways in which support is conditional.

The research and policy background to the SCD suggests a preconceived outcome for the dialogue. The SCD emerged out of a recommendation of the UK Stem Cell Initiative (UKSCI) to take into account public attitudes by engaging the UK public in a dialogue on the ethical issues surrounding the sources and uses of embryonic stem cell lines, the use of animal experimentation, and the benefits and risks of stem cell therapies [7]. The UKSCI's terms of reference express a clear mandate for stem cell research that involves developing a 10-year vision for UK stem cell research that seeks to make the UK the most scientifically and commercially productive location for this activity over the coming decade. The public dialogue also coincided with the Commons debate of the Human Fertilisation and Embryology Bill that provides for revised and updated legislation on assisted reproduction and for changes to the regulation and licensing of embryo use in research and therapy. In this context, the consensual outcome of “high levels of public support” potentially evokes pressures to achieve legitimacy for, and trust in, policy commitments in process or already decided.

Against this background, competing rationales for representing the public infused the SCD's methodology, process, and reporting. The SCD employed a statistical approach to obtain a representative sample of public views. Two hundred participants were recruited according to the demographic profile of the workshop locations to reflect quotas set for age, socioeconomic status, and ethnicity. Attitudes about stem cells were also screened to ensure the sample was broadly representative of public attitudes profiled in the results of a BMRB UK-wide omnibus survey (*n* = 1,000) which reported that 73% approved of stem cell research and 76% approved of the use of embryos in such research. Although “testing” the representativeness of participants' attitudes is a standard method [8],[9], it aggregates public views on risks and benefits to produce a majority view that can be summed up statistically, rather than creating conditions that allow for disparate perspectives in the substantive content of the dialogue.

Our observations and participant feedback question whether such conditions were created in the SCD. In response to open-ended questions in the evaluation questionnaire, one participant commented, “I suspect the people in the workshop are not really representative of the population as a whole”, while another was heard to remark in a break-out group, “I don't feel it is a realistic representation of how people feel”. Although the first comment appears to reflect the kind of statistical representativeness that we have queried, it can also be interpreted in context to mean a concern for the lack of diversity in societal perspectives articulated at the workshops.

The sponsors considered the integration of stakeholder and public voices to be one of the strengths of the SCD's methodology. In principle, such a framework could help elicit the implicit ethical assumptions of scientific and non-scientific positions, and facilitate open dialogue. Except, BMRB used findings from preceding telephone interviews with 49 stakeholders—broadly categorised as research scientists, clinicians, social scientists, ethicists, commercial and pharmaceutical organisations, religious and faith groups, medical charities, pro-life groups, funders, government, and regulators—to structure the public's deliberations in the workshops. Thus, participants were carved out at the outset into “stakeholders” and the “public” and were engaged differently. The artificial separation of “stakeholders” and the “public”—and the presumption that the public do not have an equivalent stake in the technology—meant that diverse perspectives, visions, and values could not emerge *through* the process of dialogue. Instead, the structure promoted deficit notions of experts as bearers of purely “scientific” information and the public as bearers of purely “value” commitments, creating a hierarchy that hindered genuine deliberation—in keeping with instrumental ends, such as acquiring public understanding or acceptance.

During the dialogue *process*, we observed that minority views were welcomed by the facilitators, who often took time to explore why such views were held. In spite of this encouragement, one participant in London, who believed her opinion to be contrary to other views presented, did not feel that she had the power or authority to be heard. Likewise, a Cardiff participant was reluctant, until repeatedly pressed by a facilitator, to express her opinion that human embryonic stem cell research was morally wrong. Although a few participants repeatedly interrupted or challenged the perspectives of others in the London workshops, disagreements were rare and generally amicable for such an ethically complex topic. Such disagreements, when they did occur, often centred on differing religious convictions or personal experiences with family members or friends. Participants seemed satisfied rather than frustrated with the process, although one or two participants in some workshops did leave for unknown reasons. BMRB acknowledged that this was unfortunate, noting that there were some participants with contrary views in the first workshops, distinguished along religious and cultural lines, who had subsequently fell silent or dropped out (interview, 16 December 2008).

The homogeneity of responses appears to have been shaped by the role played by experts in framing the discussion. Framing played a significant role in bounding the discussions as participants showed a strong tendency to follow and explore the main issues raised in the experts' presentations. We noted significant variations in the responsiveness of participants to particular experts who were more effective communicators. We also observed considerable homogeneity among the general views and attitudes of the scientists and clinicians, save for embryonic stem cell scientists and adult stem cell scientists. We observed that the scientists/clinicians were typically in favour of stem cell science and the ethicists/social scientists were generally reluctant to criticise it. There was an absence of experts willing to discuss the problems that have already been encountered with stem cell research and regenerative medicine (e.g., the fact that we are still at the early stages of development for cell therapies for many diseases) and other novel therapeutics (e.g., gene therapies, xenotransplantation) and the potential problems we are likely to encounter in the future (e.g., the logistical and procedural difficulties that will be involved in translating stem cell science into clinical applications).

The lack of alternative or more critical/sceptical perspectives of counter-experts (e.g., advocacy/pro-life group, religious group, journalistic, or National Health Service viewpoints) limited the range of participants' discussion and increased the potential for obtaining positively biased indications of public approval and acceptance. Counter-experts were mostly invisible in the public workshops, as they were defined in terms of religious/faith groups and pro-life groups and consigned to the external stakeholder group. Hence, the SCD can be criticised in the sense that it did not create conditions for substantive disagreements or counter perspectives to emerge in the dialogue process.

Tensions arising from competing rationales affected the ways in which the representativeness of the public participants was variously, and problematically, construed in the design of the SCD. The sponsors' aims and objectives, suggesting *normative* aspirations, aimed to create an “improved environment for dialogue between scientists, science policy makers, other stakeholders and diverse publics” (an anticipated outcome listed in the unpublished invitation to tender for the evaluation) that, initially at least, engaged the public as *subjects* of the dialogue. However, the SCD methodology limited participants' opportunities to introduce alternative frames in the *substantive* content of the dialogue, as it was structured around the predetermined topic guides and the expert presentations in the workshop. In this sense it cannot be said that the public was convincingly engaged as *architects* (or framers) of the dialogue. Finally, *instrumental* pressures exerted by the broader policy and research context, and by the artificial separation (reinforcing deficit conditions for public acceptance and limiting conditions for dissent) then integration (to create a seemingly consensual verdict on stem cell research) of public and stakeholder perspectives, suggest a strong possibility that the public was engaged as *objects* of the dialogue.

## Lessons for Public Engagement

In an earlier Perspective in this series, Stirling [3] recognises that the way to achieve a new enlightened democratic approach is not through procedural design but in the creation of a “dynamic new political arena—in which reasoned scepticism is as valued in public debates about technology as it is in science itself”. Yet the challenges of doing this in practice remain considerable.

We hypothesised that representativeness of participation, understood as manifest in a diversity of perspectives, may not necessarily confer representativeness on the outcome. And from a democratic perspective, an outcome that fails to reflect a representative range of views on stem cells renders the SCD a failure. But how and under what conditions competing rationales for engagement emerge aren't always, if ever, predictable, despite the best intentions of the organizers or sponsors. As a result, plans to mitigate such occurrences are likely to fail.

Thus, rather than focus on mending the broken process of PE, we would do better to focus on why PE is important in a democratic society. A possible way out of this democratic dilemma is one in which the public is principally engaged as the *architect/s* (rather than only as the *subject* or *object*) of these dynamic political arenas. Only in that way can the substantive conditions for uncertainty, complexity, and contingency be sustained and strengthened against the desire for predetermined outcomes and institutional pressures. In this sense it is useful to redefine the purpose of PE, not as a structured process in which initial conditions are established through a defined methodology that generates desired outcomes, but as an emergent process in which outcomes—in form, content, and number—are inherently uncertain, reflecting the indeterminate nature of public interactions. Accordingly, PE motivated by substantive and normative imperatives, undertaken as one element of a wider process of technology assessment, is more likely to fulfil the democratic ideals of PE.

## Abbreviations

BBSRC: Biotechnology and Biological Sciences Research Council
BMRB: British Market Research Bureau
MRC: Medical Research Council
PE: public engagement
SCD: Stem Cell Dialogue
UKSCI: UK Stem Cell Initiative

## References

[1] BBSRC (2008) Largest ever stem cell ‘dialogue’ provides insight into public attitudes [press release]. Available: http://www.bbsrc.ac.uk/news/archive/2008/081217-pr-stem-cell-dialogue.aspx. Accessed 19 March 2012.

[2] Marris C, Rose N (2010) Open engagement: exploring public participation in the biosciences. PLoS Biol 8 (11) e1000549 doi:10.1371/journal.pbio.1000549. 21151343 10.1371/journal.pbio.1000549 PMC2994659

[3] Stirling A (2012) Opening up the politics of knowledge and power in bioscience. PLoS Biol 10 (1) e1001233 doi:10.1371/journal.pbio.1001233. 22235193 10.1371/journal.pbio.1001233 PMC3250508

[4] Fiorino D (1989) Environmental risk and democratic process: a critical review. Columbia Journal of Environmental Law 14: 501–547.

[5] Rowe G, Frewer L (2000) Public participation methods: a framework for evaluation. Sci Technol Human Values 25 (1) 3–29.

[6] Delgado A, Kjolberg KL, Wickson F (2010) Public engagement coming of age: from theory to practice in STS encounters with nanotechnology. Public Underst Sci 20 (6) 829–845.

[7] UK Stem Cell Initiative. (2005) UK Stem Cell Initiative: report and recommendations. Available: http://www.advisorybodies.doh.gov.uk/uksci/uksci-reportnov05.pdf. Accessed 4 December 2008.

[8] Rowe G, Horlick-Jones T, Walls J, Pidgeon N (2005) Difficulties in evaluating public engagement initiatives: reflections on an evaluation of the UK GM Nation? public debate about transgenic crops. Public Underst Sci 14 (4) 331–352.

[9] Horlick-Jones T, Walls J, Rowe G, Pidgeon N, Poortinga W, et al (2006) On evaluating the GM nation? Public debate about the commercialisation of transgenic crops in Britain. New Genet Soc 25 (3) 265–288.

[10] Horlick-Jones T, Walls J, Rowe G, Pidgeon N, Poortinga W, et al.. (2007) The GM debate: risks, politics and public engagement. Abingdon: Routledge.

